# A COX‐2 Inhibitor Does Not Interfere With the Bone‐Protective Effects of Loading in Male Mice With Arthritis

**DOI:** 10.1002/jbm4.10751

**Published:** 2023-05-08

**Authors:** Suchita Desai, Jianyao Wu, Karin Horkeby, Maria Norgård, Claes Ohlsson, Sara H Windahl, Cecilia Engdahl

**Affiliations:** ^1^ Department of Laboratory Medicine, Division of Pathology, Karolinska Institutet Karolinska University Hospital Huddinge Sweden; ^2^ Sahlgrenska Osteoporosis Centre, Centre for Bone and Arthritis Research, Department of Internal Medicine and Clinical Nutrition Institute of Medicine, Sahlgrenska Academy, University of Gothenburg Gothenburg Sweden; ^3^ Centre for Bone and Arthritis Research, Department of Rheumatology and Inflammation Research Institute of Medicine, Sahlgrenska Academy, University of Gothenburg Gothenburg Sweden

**Keywords:** ANTIGEN‐INDUCED ARTHRITIS, BONE MICROARCHITECTURE, COX‐2 INHIBITOR, MECHANICAL LOADING, NSAID, PERIARTICULAR BONE LOSS

## Abstract

Mechanical loading enhances bone strength and counteracts arthritis‐induced inflammation‐mediated bone loss in female mice. It is unknown whether nonsteroidal anti‐inflammatory drugs (NSAIDs; eg, COX‐2 inhibitors) can reduce inflammation without affecting the loading‐associated bone formation in male mice. The aim of this study was to investigate if loading combined with a COX‐2 inhibitor (NS‐398) could prevent arthritis‐induced bone loss and inflammation in male mice. Four‐month‐old male C57BL/6J mice were subjected to axial tibial mechanical loading three times/week for 2 weeks. Local mono‐arthritis was induced with a systemic injection of methylated bovine serum albumin on the first day of loading, followed by a local injection in one knee 1 week later. The arthritis induction, knee swelling, bone architecture, and osteoclast number were evaluated in the hind limbs. C‐terminal cross‐links as a marker for osteoclast activity was measured in serum. Compared with loading and arthritis alone, loading of the arthritic joint enhanced swelling that was partly counteracted by NS‐398. Loading of the arthritic joint enhanced synovitis and articular cartilage damage compared with loading alone. Loading increased cortical bone and counteracted the arthritis‐induced decrease in epiphyseal bone. NS‐398 did not alter the bone‐protective effects of loading. C‐terminal cross‐links, a bone resorption marker, was increased by arthritis but not loading. In conclusion, loading prevented arthritis‐induced epiphyseal and metaphyseal bone loss, and NS‐398 reduced knee swelling without affecting the bone‐protective effects of loading. If our results can be extrapolated to the human situation, specific COX‐2 inhibitors could be used in combination with loading exercise to prevent pain and swelling of the joint without influencing the bone‐protective effects of loading. © 2023 The Authors. *JBMR Plus* published by Wiley Periodicals LLC on behalf of American Society for Bone and Mineral Research.

## Graphical abstract

(1) Mechanical loading prevents arthritis‐induced bone loss in male mice. (2) The COX‐2 inhibitor NS‐398 reduces swelling in the arthritis‐induced mouse model. (3) The COX‐2 inhibitor NS‐398 does not interfere with the loading response.

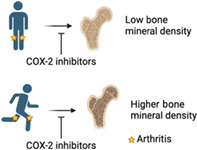

## Introduction

Rheumatoid arthritis (RA) is an autoimmune disease characterized by inflammation in the joint, which results in joint destruction and bone loss.^(^ ^1^ ^)^

The inflammation process increases the expression of cytokines both locally and systemically that creates an imbalance in bone remodeling favoring osteoclast‐mediated bone resorption over osteoblast‐mediated bone formation. Bone loss is a pathological hallmark in RA, and there are three forms of bone loss described: local bone erosions directly mediated from the site of inflammation; periarticular trabecular bone loss adjacent to, but not in direct contact with, the affected joint; and systemic osteoporosis mainly due to the autoimmune induction.^(^ ^2^, ^3^, ^4^, ^5^ ^)^ Bone destruction has an early onset in the disease course and is associated with impaired physical function in the affected patients.^(^ ^6^ ^)^

Mechanical loading of bones has been shown to improve bone mass, architecture, and strength.^(^ ^7^ ^)^ We have previously described that in vivo mechanical loading plays an essential role in preventing arthritis‐mediated bone loss in a female mouse model of mono‐arthritis.^(^ ^8^ ^)^ Physical activity has numerous health benefits also in patients with RA, including improved physical function and mobility and reduced rheumatoid cachexia and fatigue.^(^ ^9^, ^10^ ^)^ Bone‐loading physical activity in the case of arthritis would help in reducing bone loss. However, healthcare providers and patients are hesitant to promote/perform bone‐loading activity in active arthritis because of joint pain.^(^ ^11^ ^)^ Therefore, it would be beneficial to combine bone‐loading physical activities with nonsteroidal anti‐inflammatory drugs (NSAIDs) that reduce both inflammation and pain.

Different NSAIDs have been used to ease the pain and reduce the inflammation that accompany arthritis. Cyclooxygenase‐2 (COX‐2) inhibitors are a group of selective NSAIDs that target the COX‐2 enzyme that increases during loading and inflammation. In postmenopausal women, daily treatment with a COX‐2 inhibitor increased total hip bone mineral density (BMD), whereas it was decreased in men.^(^ ^12^ ^)^ COX‐2 signaling is upregulated early in response to loading and is involved in regulating bone's adaptive response to loading.^(^ ^13^, ^14^ ^)^ However, female mice exposed to a daily dose of the specific COX‐2 inhibitor NS‐398 in combination with repeated sessions of mechanical loading showed that COX‐2 inhibition did not alter the loading response.^(^ ^15^ ^)^ Whether a COX‐2 inhibitor would affect the loading response in male mice is unknown.

Because arthritis‐associated inflammation in the joint causes discomfort, pain sensation, and bone loss, the aim of this study was to investigate if (I) NS‐398 could reduce the inflammation induced by loading and arthritis, (II) mechanical loading can prevent arthritis‐mediated bone loss also in a male mouse model of mono‐arthritis, and (III) NS‐398 would inhibit the bone‐forming and ‐preserving effects of mechanical loading in male mice. We hypothesized that mechanical loading could prevent the arthritis‐induced bone loss in male as in female mice and that NS‐398 will ameliorate the inflammation induced by loading and arthritis, but NS‐398 will not decrease the bone‐forming and ‐preserving effects of mechanical loading.

## Materials and Methods

### Animals and immunizations

Male C57BL/6J mice were obtained from Janvier (Le Genest‐Saint‐Isle, France), housed at the Sahlgrenska Academy, Laboratory of Experimental Biomedicine in Gothenburg, Sweden, and fed pellet diet *ad libitum* (Teklad Diet 2826, Madison, WI, USA). The investigator was blinded during allocation to the cages and further measurements. The treatment groups were mixed in cages with 5 mice per cage. The study was approved by the animal Ethics Committee in Gothenburg (6074/17).

Sample size analysis was performed using the G*power software for the cortical area and thickness and periosteal circumference (Peri C) from a previous study.^(^ ^16^ ^)^ With an alpha value of 0.05, power of 80%, and the calculated Cohen's *d* effect size of 2 for cortical area (Ct.Ar), 2 for cortical thickness (Ct.Th), and 1 for Peri C, the calculated sample size for the different parameters were as follows: Ct.Ar (*N* = 5), Ct.Th (*N* = 5), Peri C (*N* = 6), indicating that an *N* = 6 would be required in our study to reach significance.

Sixteen‐week‐old C57BL/6 male mice were allocated only for the *ex vivo* strain measurements, which were performed before the start of the experiment (*N* = 6). Siblings to these mice were randomly divided into the following treatment groups based on the baseline weight: (i) *loading*: intra‐articular (IA) PBS injection (left side) and IA PBS injection loading (right side) (*N* = 6); (ii) *methylated bovine serum albumin* (*mBSA*): IA PBS injection (left side) and IA mBSA injection (right side) (*N* = 6); (iii) *mBSA + loading*: IA PBS injection (left side), IA mBSA injection and loading (right side) (*N* = 8); (iv) *loading + NS‐398*: same as in 1+ NS‐398 (*N* = 6); (v) *mBSA +* NS‐398: same as in 2+ NS‐398 (*N* = 7); (vi) *mBSA + loading +* *NS‐398*: same as in 3+ NS‐398 (*N* = 8). The mice were weighed 19 days before the first loading and thereafter three times per week in conjunction with the loading sessions.

As controls for the mBSA and C‐terminal cross‐links (CTX) ELISAs, serum from naïve C57BL/6J mice were taken from another set of 10‐week‐old male mice (*N* = 7).

### Antigen‐induced mono‐arthritis (AIA)

Arthritis was induced with antigen‐induced mono‐arthritis (AIA), a method previously used for detecting arthritis‐induced bone loss in 12‐week‐old female mice.^(^ ^17^ ^)^ All mice were immunized systemically on day 1 (loading session 1; Supplemental Fig. S1 *A*), by an intradermal injection of 0.1 mg mL^−1^ mBSA (Sigma‐Aldrich, Sollentuna, Sweden) emulsified in equal amounts of complete Freund's adjuvant (CFA) (Sigma‐Aldrich) containing 1 mg mL^−1^ heat‐inactivated *Mycobacterium tuberculosis*. The primary systemic immunization was followed by induction of a local inflammation by an IA injection of mBSA in one knee (arthritic knee) and PBS in the contralateral knee (non‐arthritic knee) on day 8 (loading session 4). The PBS‐injected side was used as contralateral internal control, as previously described.^(^ ^17^ ^)^ The induction of mono‐arthritis inflammation was monitored using a caliper to measure the diameter of the knee.

### Ex vivo strain measurements

Before in vivo loading, the magnitude of the mechanical strain was established on a subset of the animals by using an ex vivo strain gauging protocol as described previously.^(^ ^18^ ^)^ In short, mice were euthanized, and single‐element gauges (EA‐06‐015DJ‐120, Vishay Precision Group, Malvern, PA, USA) were placed on the crano‐medial side at 37% length from the proximal end of the tibia. A range of varying loads between 6 to 18 N was applied to measure the strain (*N* = 6). The 3100 Electroforce test instrument was used to apply the load and a PCI‐box was used to measure the strain (Bose Corporation, currently available at TA Instruments, Eden Prairie, MN, USA). A load‐strain curve was plotted to derive the peak strain value (Supplemental Fig. S1 *B*). A peak strain corresponding to 2400 με was reached at 13.5 N and chosen for the experiment.

### In vivo loading

Axial tibial mechanical loading was performed as described previously^(^ ^18^, ^19^ ^)^ (Supplemental Fig. S1 *A*). A load of 13.5 N was applied to the right tibia using the same loading equipment as for the ex vivo strain measurements (3100 Electroforce test instrument, Bose Corporation, currently available at TA Instruments) on days 1, 3, 6, 8, 10, and 12 under the influence of anesthesia with isoflurane (Baxter Medical AB, Kista, Sweden). The non‐loaded animals received the same anesthetic treatment. Forty cycles/day of compressive load was applied with a rest period of 10 seconds between each cycle in a trapezoidal waveform. Before each loading session, a subcutaneous injection of either the COX‐2 inhibitor NS‐398 (Sigma‐Aldrich), at a concentration of 5 mg/kg/d dissolved in NaCl, or only the vehicle NaCl was administered.

The swelling in the knee joint was measured using a digital caliper with a 0.03 mm accuracy (RS PRO, RS, Shanghai, China). The remaining analyses were performed after euthanization on day 14. No adverse events occurred in any of the treatment groups.

### X‐ray analyses

Dual‐energy‐X‐ray absorptiometry (DXA) analyses were performed to assess areal bone mineral density (aBMD), in the epiphyseal region of the proximal tibia close to the affected joint as previously described by Sehic and colleagues^(^ ^20^ ^)^ and in a fixed area 5 mm from the growth plate in the diaphyseal tibia (Supplemental Fig. S1 *C*) using Faxitron UltraFocus DXA of 40 kV and 0.28 mA for 2.53 seconds with the spatial resolution of 24 μm using 2× geometric magnification (Faxitron Bioptics, Tucson, AZ, USA). The epiphyseal tibia contains mainly trabecular bone but also includes subchondral cortical bone.

High‐resolution microcomputed tomography (μCT) was performed using a Skyscan 1172 scanner (Bruker MicroCT, Kontich, Belgium). The trabecular bone of the tibia was investigated distal to the growth plate at 0.504 mm in the metaphysis of intact knee, extending up to 0.196 mm. The cortical μCT measurements in the tibia were performed in the mid‐diaphyseal region of the tibia at 5.3 mm from the growth plate and extended longitudinally 0.196 mm in the proximal direction. The software CTvol was used for the representative 3D construction images.

### Histological examination

Knee joints were fixed in 4% formalin, decalcified in EDTA (Sigma‐Aldrich), and embedded in paraffin. Sagittal sectioning was done between the lateral and condyle of the tibia (2 μm) (Histocenter, Västra Förlunda, Sweden), and the sections were stained with hematoxylin and eosin. The inflammation and local joint destruction were graded by an examiner blinded to the interventions using a blunt 3‐grade histological scoring system (mild‐1, moderate‐2, or severe‐3) as previously described.^(^ ^8^ ^)^ Briefly, for synovitis, 1 is defined as minimal synovial hypertrophy (>two cell layer) or immune cells in the joint area, 2 as extended synovial hypertrophy or cells around the tendon junction, and 3 as synovial hypertrophy and cells within the whole joint space. For bone erosion, 1 is defined as limited destruction of the bone by immune cells in less than two spots, 2 as erosion in more than two spots or immune cells invading the marrow space in one spot, and 3 as destruction of bone in several locations or cells invading the marrow space in more than two spots. For articular cartilage damage, 1 is defined by limited areas showing cartilage destruction or cells growing in less than two spots over the bone, 2 as cartilage destruction in more than two spots or cells growing over the bone, and 3 where most of the cartilage is destroyed and the cells are located within the bone.

Medial sections of the knee were stained for cathepsin K by immunohistochemistry. In short, after removal of paraffin in xylene and rehydration in descending concentrations of ethanol (100%–70%) followed by H_2_O, the sections were blocked with 3% H_2_O_2_ for 5 minutes followed by blocking with normal goat serum (NGS) in PBST (1:5) for 20 minutes. The sections were then stained with primary rabbit anti‐cathepsin K antibody (Supplemental Table S1) diluted in PBST+1.5% NGS (1:300) for 2 hours at room temperature (RT). This was followed by incubation with biotinylated secondary goat anti‐rabbit antibody (Supplemental Table S1) diluted in PBST (1:300) for 30 minutes. Detection was performed using VectaStain ABC Kit (Vector Laboratories, Burlingame, CA, USA) followed by DAB staining. One image was taken per mouse using a Nikon (Tokyo, Japan) Eclipse E1000M, DS‐Fi1 microscope equipped with a digital camera. The images were assessed for osteoclast number, osteoclast surface/bone surface, and osteoclast number/bone perimeter between the growth plate and the articular cartilage on the proximal end of the tibia (epiphyseal region) in the medial section of the knee using Osteomeasure (OsteoMetrics, Decatur, GA, USA).

### Serum analyses

Blood was taken from the axillary vessels at termination, and serum was prepared using Multivette tubes (Sarstedt, Helsinborg, Sweden) standing in room temperature for 20 minutes followed by centrifugation. The serum samples were individually stored at –20°C. mBSA‐specific immune globulin G (IgG) was assessed in triplicate using ELISA as previously described.^(^ ^21^ ^)^ ELISA plates were coated with 0.01 mg/mL mBSA, blocked, and washed with milk powder (Carl Roth, Frankfurt, Germany) before adding serum. Bound anti‐mBSA antibodies were then detected by horseradish peroxidase (HRP)‐conjugated rabbit‐anti mouse IgG (Supplemental Table S1).

As a marker for osteoclast activity, serum levels of C‐terminal type I collagen fragments (CTX‐I) were determined with the ELISA Crosslaps Kit (Immunodiagnostic Systems, Boldon, UK).

### Statistical analysis

Statistical analysis was performed using GraphPad Prism version 9 (La Jolla, CA, USA). Normal distribution of data was assessed using Shapiro–Wilk test, and data from X‐ray analysis, serum analysis, and osteoclast histomorphometry was found normally distributed. For the X‐ray analysis and osteoclast histomorphometry, the PBS‐injected non‐loaded contralateral leg was used as an internal control in each mouse and compared with the affected knee with a Student's paired *t* test. Data for knee swelling are presented as differences from baseline in Kaplan–Meier curves. The area under the curve between the different treatment groups was assessed for the NSAID or vehicle‐treated groups separately by analysis of variance (ANOVA) followed by Tukey's multiple comparison test. The difference between the treatment groups from PBS‐injected non‐loaded contralateral leg was assessed using paired *t* test. Difference in swelling in the presence or absence of NSAIDs between days 11 and14 was assessed using unpaired *t* test. The X‐ray analysis, serum analysis and osteoclast histomorphometry were assessed for the NSAID or vehicle‐treated groups separately using ANOVA followed by a Tukey's multiple comparison test. For the ordinal scale comparison (histological scoring), the nonparametric Kruskal–Wallis test followed by Dunn's post hoc test was used to compare the different treatment groups. For the paired comparison of the histological scoring, data between the affected knee and the PBS control, Wilcoxon signed rank test was used. A *p* value ≤0.05 was considered significant.

## Results

### NS‐398 reduced swelling in the loaded arthritic knee

**Fig. 1:**
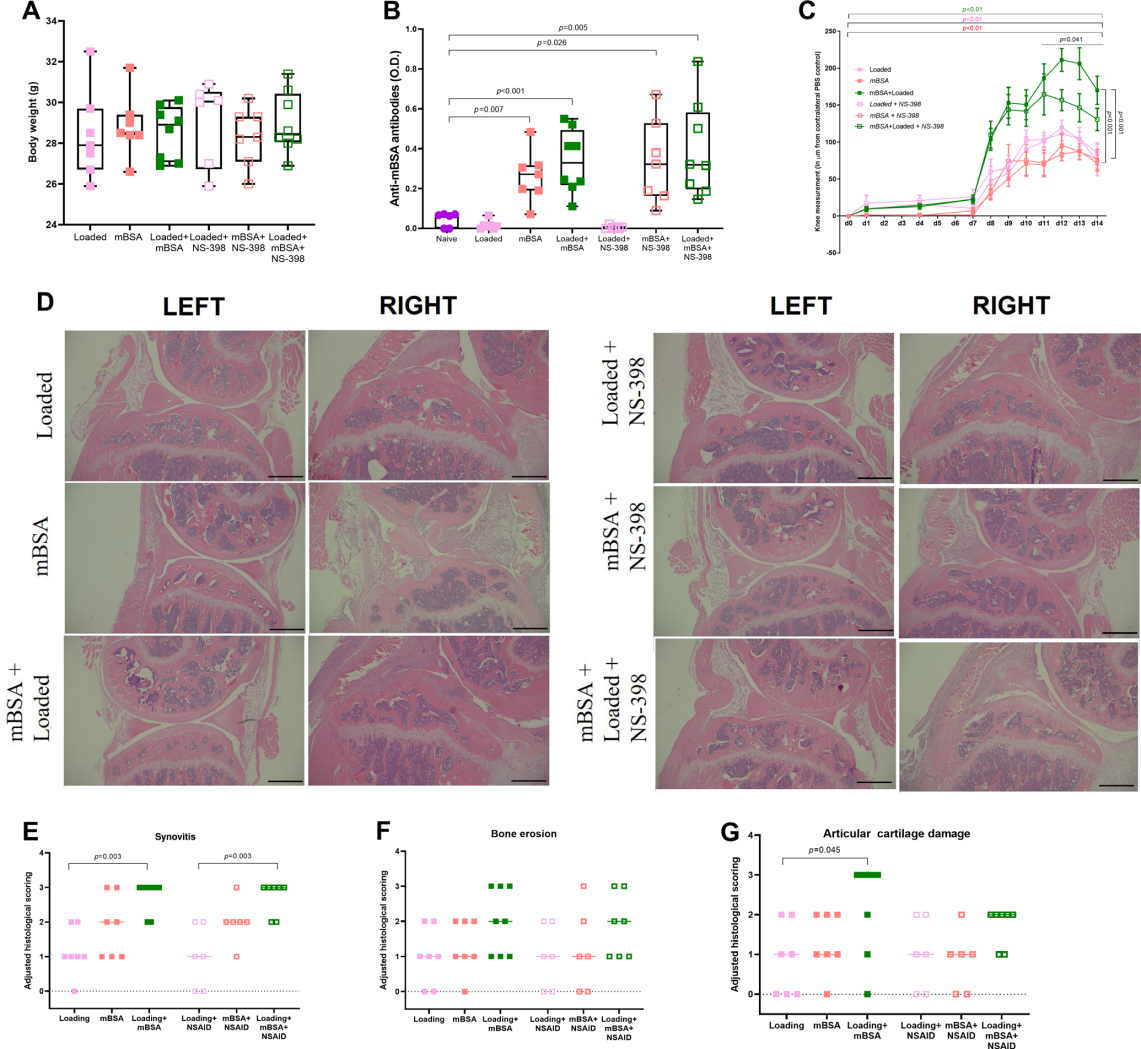
NS‐398 reduced swelling in the loaded arthritic knee. (*A*) Body weight on day 14, (*B*) anti‐mBSA antibody levels, (*C*) swelling of the knee adjusted to the contralateral PBS control, (*D*) representative images from H&E‐stained histological sections of the tibia and adjusted histological scoring of (*E*) synovitis, (*F*) bone erosion, and (*G*) articular cartilage damage are shown. (*D*) Left leg corresponds to internal control and the right leg corresponds to treatment as indicated on the left‐hand side of the images. Scale bars = 500 μm. (*A*, *B*) One‐way ANOVA followed by Tukey's multiple comparison. (*C*) Paired *t* test was performed to assess the effect of treatment compared with the PBS control (statistics marked by staple and colored *p* values over the graph). Comparison between mBSA + loading with mBSA or loading alone was performed by ANOVA followed by Tukey's multiple comparison (statistics marked by staples to the right of the graph). An unpaired *t* test was performed to assess the effect of loading + mBSA in the presence or absence of NSAIDs at days 11 to 14 (statistics marked by line over the graph). (*E*–*G*) A nonparametric Kruskal–Wallis test followed by Dunn's multiple comparison was performed. Results are shown as median, min. max. to show all points (*A*–*C*) or mean ± SEM (*E*–*G*). Each group *N* = 6–8.

Multiple occasions of sedation cause stress and can result in reduced body weight. We therefore measured body weight at the end of the experiment. There was no change in body weight at termination (Fig. 1*A*). To assess whether the induction of arthritis was successful, mBSA antibodies were measured in serum by ELISA. Serum mBSA was increased in both mBSA and the mBSA + loaded groups compared with the naïve control. This effect remained unchanged in the presence of NS‐398 (Fig. 1*B*).

Loading and mBSA induced swelling over the knee joint to the same degree compared with the contralateral PBS control. This swelling was further enhanced by the combination of the two. In contrast, NS‐398 did not affect the swelling of the joint induced by loading or mBSA but decreased the swelling in the loaded arthritic joint between days 11 and 14 (Fig. 1*C*).

Histological images and scoring of H&E‐stained sections of the knee joints showed that loading, mBSA, and loading with mBSA all induced an inflammation in the synovia, synovitis, compared with the contralateral PBS control (Supplemental Fig. S2*A*). NS‐398 did not change the increased synovitis found in the mBSA and mBSA + loading group. However, the effect of loading on synovitis was no longer significant in the presence of NS‐398 compared with the contralateral PBS control (Supplemental Fig. S2*A*). Bone erosion was increased by mBSA and loading with mBSA compared with the contralateral PBS control (Supplemental Fig. S2*B*). In the presence of NS‐398, bone erosion was still enhanced in the mBSA + loading group compared with the contralateral PBS control (Supplemental Fig. S2*B*). Treatment with mBSA in the presence or absence of loading, but not loading alone, enhanced the articular cartilage damage when compared with the PBS control (Supplemental Fig. S2*C*). In the presence of NS‐398, the cartilage damage was only enhanced in the mBSA + loading group compared with the PBS control (Supplemental Fig. S2*C*).

Synovitis was more prominent in the loaded mBSA‐treated joint compared with loading alone, and this effect remained the same in the presence of NS‐398 (Fig. 1*E*). Neither mBSA nor NS‐398 affected bone erosion when compared with loading alone (Fig. 1*F*). In addition, loading of the arthritic joint significantly increased the cartilage damage when compared with loading alone, an effect that was lost in the presence of NS‐398 (Fig. 1*G*).

### Loading prevented the arthritis‐induced decrease in epiphyseal BMD

**Fig. 2:**
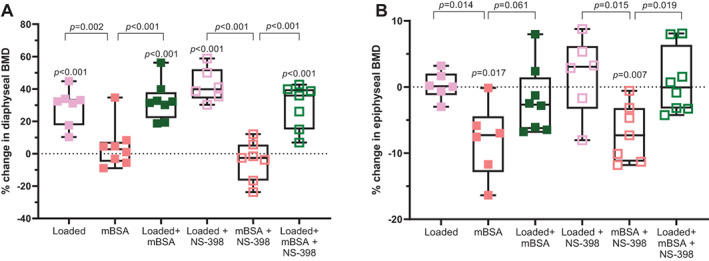
Loading reduced the arthritis‐induced subchondral bone loss. Dual‐energy X‐ray absorptiometry (DXA) analysis of (*A*) diaphyseal and (*B*) epiphyseal bone mineral density (BMD) in the proximal region of tibia, shown as percentage change relative to the contralateral PBS control leg. The dotted line indicates the contralateral PBS control. The *p* values above each treatment group indicate significant difference from PBS control. One‐way ANOVA followed by Tukey's multiple comparison test was performed. Results are shown as median, min. max. to show all points. Each group *N* = 6–8.

DXA analysis showed that loading alone and loading + mBSA increased diaphyseal BMD in tibia compared with both the contralateral PBS control and mBSA alone (Fig. 2*A*). NS‐398 did not inhibit the loading‐induced increase in cortical BMD. BMD in the epiphyseal area of the proximal tibia decreased significantly by mBSA treatment in the absence or presence of NS‐398 compared with the contralateral PBS control (Fig. 2*B*). Loading alone did not affect epiphyseal BMD but prevented the mBSA‐induced decrease in epiphyseal BMD (Fig. 2*B*). NS‐398 did not inhibit this bone‐protective effect of loading.

### Loading prevented the arthritis‐induced decrease in metaphyseal bone

**Fig. 3:**
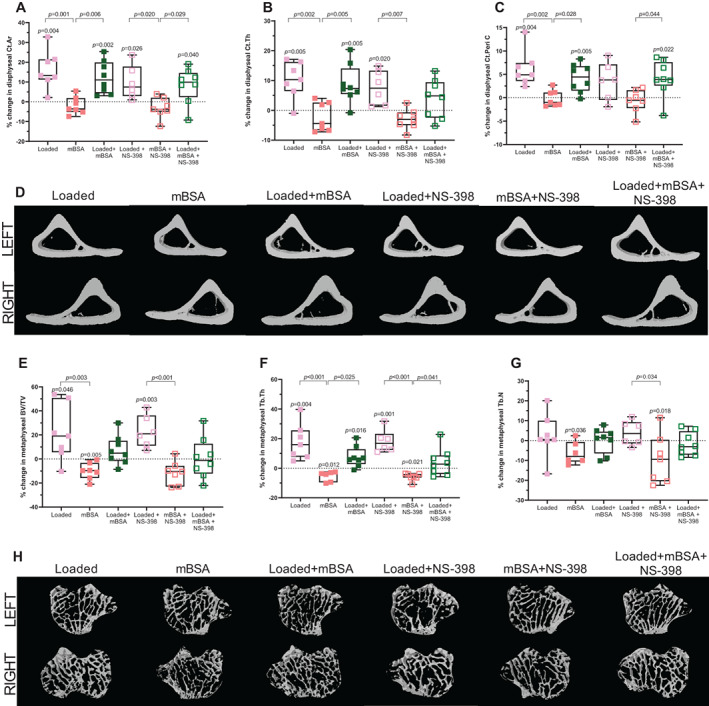
Loading prevents the arthritis‐induced bone loss also in the presence of NS‐398. Microcomputed tomography (μCT) analysis of cortical (*A*) area (Ct.Ar), (*B*) thickness (Ct.Th), and (*C*) periosteal circumference (Ct.Peri C) are shown as percentage change relative to the contralateral PBS control leg. Representative 3D images for μCT data‐cortical bone at 37% length from proximal end of the tibia. Left leg corresponds to internal control and the right leg corresponds to treatment as indicated above the images (*D*). μCT analysis of trabecular (*E*) bone volume fraction (BV/TV), (*F*) thickness (Tb.Th) and (*G*) number (Tb.N) are shown as percentage change relative to the contralateral PBS control leg. The dotted line indicates the contralateral PBS control. The *p* values above each treatment group indicate significant difference from PBS control. Representative 3D images for μCT data‐trabecular bone in the proximal metaphysis of the tibia (*H*). One‐way ANOVA followed by Tukey's multiple comparison test was performed. Results are shown as median, min. max. to show all points. Each group *N* = 6–8.

Detailed μCT measurements revealed that the diaphyseal cortical area, thickness, and periosteal circumference were all increased in response to loading, but not mBSA treatment, when compared with the contralateral PBS control (Fig. 3*A*–*C*). In the proximal metaphysis, the tibial bone volume fraction (BV/TV) and trabecular thickness were increased by loading alone and decreased by mBSA treatment alone compared with the contralateral PBS control (Fig. 3*E*, *F*). Loading prevented the mBSA‐induced reduction in BV/TV and trabecular thickness compared with the PBS control. In addition, trabecular thickness was higher in the loading + mBSA group compared with mBSA alone. In contrast, trabecular number was not altered by any treatment compared with the contralateral PBS control (Fig. 3*G*). NS‐398 did not inhibit this bone‐forming and bone‐protective effect of loading on cortical and trabecular bone (Fig. 3).

### Osteoclast activity is increased in the presence of arthritis but not loading

**Fig. 4:**
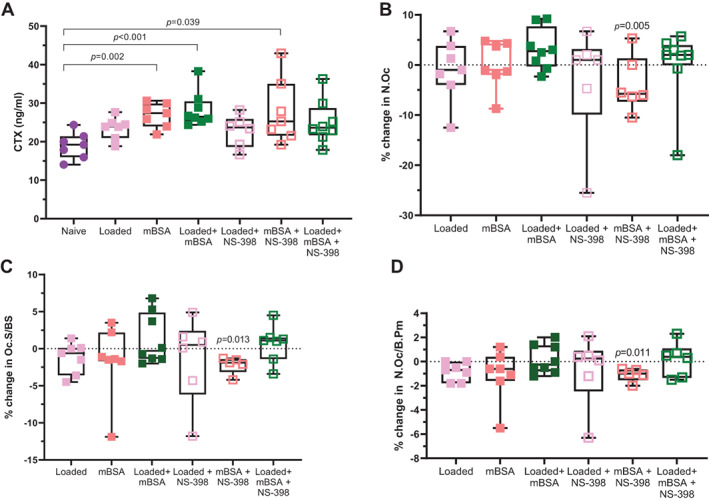
C‐terminal collagen type‐1 cross‐links are enhanced by arthritis but not loading. (*A*) Serum analysis of C‐terminal collagen type‐1 cross‐links (CTX‐1) is shown. Subchondral histomorphometric analysis of (*B*) number of osteoclasts (N.Oc), (*C*) osteoclast surface/bone surface (Oc.S/BS), and (*D*) number of osteoclasts per bone perimeter (N.Oc/B.Pm) shown as percentage change relative to the contralateral PBS control leg. The dotted line indicates the contralateral PBS control. One‐way ANOVA followed by Tukey's multiple comparison test. Results are shown as median, min. max. to show all points. Each group *N* = 6–8.

To investigate whether the effects found in bone mass and architecture were attributable to altered osteoclast activity, we measured the bone resorption marker CTX in serum. The serum CTX‐1 level was not changed in response to load. However, it was increased by mBSA, in the absence or presence of loading, compared with the naïve group (Fig. 4*A*). The same trend was found in the presence of NS‐398, but the increase in the CTX found in the loaded mBSA group compared with naïve control was absent in the presence of NS‐398. To investigate whether the effects found in bone mass and architecture were attributable to altered osteoclast number or surface, we performed static histomorphometry. The osteoclast number, osteoclast surface per bone surface, and number of osteoclasts per bone perimeter were not changed by loading, arthritis, or the combination thereof when compared with the contralateral PBS control. Cotreatment with NS‐398 did not alter any of these osteoclast parameters in response to loading or mBSA + loading, but they decreased by mBSA treatment compared with the contralateral PBS control (Fig. 4*B*–*D*). In contrast, treatment with NS‐398 did not alter the effect of loading, mBSA, or the combination of the two on the osteoclast number, osteoclast surface per bone surface, or number of osteoclasts per bone perimeter (Fig. 4*B*–*D*).

## Discussion

Load‐bearing exercise is a key regulator of bone architecture and strength, and thus regular exercise is one of the recommended treatments to maintain bone strength and quality in all individuals, including patients with arthritis. However, because of pain and inflammation, patients might refrain from exercise.^(^ ^11^, ^22^, ^23^ ^)^ NSAIDs could be used to reduce pain and inflammation associated with arthritis and exercise. However, as NSAIDs inhibit COX‐2, and COX‐2 is one of the early upregulated genes in response to load,^(^ ^15^ ^)^ there is a possibility that treatment with NSAIDs during exercise may lead to reduced loading response depending on the type and dose of NSAID used.^(^ ^14^, ^16^ ^)^ A decrease in swelling over the joint was observed in collagen‐induced arthritis, a chronic polyarthritis model, after treatment with the same NSAID as we used,^(^ ^24^ ^)^ but no investigations were performed in mono‐arthritis with mBSA. Our model of mono‐arthritis with mBSA increases periarticular bone loss,^(^ ^17^ ^)^ whereas mechanical loading in the presence of mono‐arthritis with mBSA improves cortical and trabecular bone parameters in young adult female mice.^(^ ^8^ ^)^ In this study, we investigated if (I) NS‐398 could reduce the inflammation induced by loading and arthritis, (II) mechanical loading can prevent arthritis‐mediated bone loss also in a male mouse model of mono‐arthritis, and (III) NS‐398 would inhibit the bone‐forming and ‐preserving effects of mechanical loading in male mice.

There was limited swelling in the loaded knees during the first week of loading. During the second week of loading, loading, and arthritis alone resulted in further swelling to a similar extent of the knee joint compared with the contralateral PBS‐treated joints. The combination of loading with mBSA treatment resulted in a pronounced swelling when compared with contralateral PBS‐treated joints. These results were consistent with our previous study in female mice showing that loading of the arthritic leg increased the swelling further compared with arthritis induction alone.^(^ ^8^ ^)^ The COX‐2 inhibitor NS‐398, known to reduce the inflammation and joint swelling,^(^ ^25^, ^26^ ^)^ was able to reduce the swelling in the loaded arthritic joints, indicating that the inhibitor was effective in reducing the swelling caused by arthritis and loading.

Histological assessment showed that all interventions resulted in clinical signs of arthritis: synovitis as well as bone and cartilage destruction when compared with the contralateral PBS‐treated joints. Synovitis, bone erosion, and cartilage damage were increased in response to arthritis induction alone and loading of the arthritic leg.^(^ ^27^ ^)^ Loading of the arthritic leg further increased the synovitis and articular cartilage destruction compared with loading, but not mBSA alone, indicating that loading alone had less of a role in the damage observed in the joint. Similar effects were found in our previous study involving female mice.^(^ ^8^ ^)^ Addition of NS‐398 prevented the increase in articular cartilage damage only.

Radiological examination showed that arthritis‐induced bone loss was observed in trabecular bone close to the knee joint, the site of the intra‐articular injection. There was no bone loss in the diaphyseal cortical bone because of arthritis induction. This pattern mimics the human situation where most bone loss is found in trabecular bone close to the joint in RA patients.^(^ ^4^ ^)^ In contrast, loading prevented the arthritis‐induced decrease in BMD at the epiphyseal bone and increased BMD in diaphyseal bone. Interestingly, although COX‐2 is one of the earliest upregulated genes in response to loading,^(^ ^15^ ^)^ addition of the specific COX‐2 inhibitor NS‐398 did not affect the loading response at any location. This contrasts with two previous studies in female rats showing that NS‐398 reduced the endocortical bone formation in response to loading.^(^ ^28^, ^29^ ^)^ There could be several reasons for this discrepancy. First, the route of administration was different (oral versus intraperitoneal). Second, different species were used. Third, previous studies have assessed the change in bone formation at the endocortex, whereas we have assessed cortical BMD and bone area and trabecular bone volume fraction. However, our study is in line with another study using the same dose of NS‐398 in female mice showing that a single dose of NS‐398 and loading did not interfere with the bone‐forming effects of loading.^(^ ^15^ ^)^ Therefore, we conclude that a COX‐2 inhibitor does not interfere with the bone‐forming effects of loading, at least in mice, and a COX‐2 inhibitor may be used in concert with loading to prevent arthritis‐induced bone loss, inflammation, and pain.

In line with an increased bone mass, there was no increase in serum CTX levels or osteoclast number/surface in response to load. In contrast, serum CTX was increased in the arthritic mice in the presence or absence of loading. This increase correlated with a decrease in epiphyseal BMD and metaphyseal trabecular BV/TV ratio found in arthritic legs in the absence of loading. However, the number of osteoclasts did not correlate with the increase found in serum CTX levels. This is in contrast to our previous study in female mice, where the osteoclast number was positively correlated with the CTX values.^(^ ^8^ ^)^ We speculate that the increase in CTX observed in male mice in our present study may be attributable to an increase in activity, but not number, of the existing osteoclasts. In line with the idea, Mun and colleagues showed that 8‐week‐old female mice have a higher osteoclast number than male mice and that an inflammatory environment enhances the activation of the osteoclast precursors more in female than in male mice.^(^ ^30^ ^)^ The addition of NS‐398 did not alter the effect of loading, mBSA, or the combination of the two on any osteoclast parameter. Other studies have shown that the type and the dose of NSAIDs as well as the stage of inflammation can affect osteoclast formation and activity, which contributes to the disease progression.^(^ ^14^ ^)^ Different strains have been reported for human physical activities. The strain obtained during the loading regimen used in this study has been obtained in response to jumping.^(^ ^31^ ^)^ Depending on the severity of the arthritis, the load that could be placed on the joints may vary. Thus, further research is needed to determine the dose and exercise regimen needed, depending on the stage of the disease, to optimize bone protection and minimize pain and damage to the joint, when transferred to the human situation.

Our study has limitations. First, the model inducing mono‐arthritis is not a model of RA, which is an autoimmune disease, and the degree of the symptoms varies depending on the stage of the disease. Our protocol mimics periarticular osteopenia with mono‐arthritis and local bone reduction. Second, we only used one dose of the NS‐398. It is possible that a higher dose of and/or another NSAID could result in a further reduction in inflammation and joint swelling. Third, we used a higher load compared with Liphardt and colleagues,^(^ ^8^ ^)^ which may have caused more damage to the joint.

Our study also has advantages. The model of mono‐arthritis is the only currently available method that facilitates investigation of local bone reduction, and axial mechanical loading of the tibia enables investigation of load‐induced bone formation without involvement of muscle‐associated effects of exercise in mice.^(^ ^7^ ^)^ NS‐398 is a specific COX‐2 inhibitor; thus, we were able to investigate the effects of COX‐2 inhibition without affecting COX‐1.^(^ ^26^ ^)^ We show here that the use of this COX‐2 inhibitor reduced the swelling and damage in the cartilage associated with arthritis and that loading, in the presence of a specific COX‐2 inhibitor, still reduces the arthritis‐induced bone loss.

We conclude that a low dose of the COX‐2 inhibitor NS‐398 does not interfere with the bone‐forming effects of loading. In addition, loading in combination with NS‐398 prevents arthritis‐induced bone loss and inflammation in male mice. If our results can be translated to the human situation, COX‐2 inhibitors could be used in combination with exercise to reduce pain and inflammation without intefering with exercise preventing of arthritis‐mediated bone loss.

## Author Contributions

**Suchita Desai:** Conceptualization; data curation; formal analysis; investigation; methodology; validation; visualization; writing – original draft; writing – review and editing. **Jianyao Wu:** Data curation; formal analysis; methodology; writing – review and editing. **Karin Horkeby:** Data curation; methodology; writing – review and editing. **Maria Norgård:** Methodology; writing – review and editing. **Claes Ohlsson:** Methodology; resources; software; supervision; writing – review and editing. **Sara H Windahl:** Conceptualization; data curation; formal analysis; funding acquisition; investigation; methodology; project administration; resources; software; supervision; validation; visualization; writing – review and editing. **Cecilia Engdahl:** Conceptualization; data curation; formal analysis; funding acquisition; investigation; methodology; project administration; resources; software; supervision; validation; visualization; writing – review and editing.

## Author Roles

Study design: SD, CO, SHW, CE. Study conduct: SD, JW, KH, MG, SHW, CE. Data collection: SD, JW, KH, MG, SHW, CE. Data analysis: SD, JW, CO, SHW, CE. Data interpretation: all authors. Drafting manuscript: SD, SHW, CE. Revision manuscript: all authors.

## Disclosures

SD, JW, KH, MN, SHW, and CE declare no conflicts of interest. CO is an applicant on patents filed in the field of the effect of probiotics in osteoporosis.

### Peer Review

The peer review history for this article is available at https://www.webofscience.com/api/gateway/wos/peer-review/10.1002/jbm4.10751.

## Supporting information


**Supplemental Fig S1.** Timeline of the experiment and strain gauging. (*A*) Local mono‐arthritis was induced with a systemic injection of mBSA on the first day of loading, followed by a local injection in one knee 1 week later. The mice were loaded for three sessions per week for 2 weeks. Each loading session was accompanied by a subcutaneous injection of NS‐398 (5 mg/kg/d) or vehicle. (*B*) The relationship between peak dynamic load and strain at the gauge site as calculated using linear regression analysis. Values are presented as mean ± SEM (*N* = 6). (*C*) Representative image of the area for epiphyseal and diaphyseal measurement in part of tibia.
**Supplemental Fig S2.** Histological scoring of (*A*) synovitis, (*B*) bone erosion, and (*C*) articular cartilage damage is shown. Wilcoxon signed rank test was performed. Results are shown as median, min. max. to show all points; each group *N* = 6–8.
**Supplemental Table S1.** Antibodies Supplemental Table.
