# Supplementary material for: A COX‐2 Inhibitor Does Not Interfere With the Bone‐Protective Effects of Loading in Male Mice With Arthritis
Source: JBMR Plus. 2023 May 8;7(7):e10751. doi: 10.1002/jbm4.10751 (PMC10339087; doi:10.1002/jbm4.10751)
Supplement: Supplementary file 1 — Supplemental Fig S1. Timeline of the experiment and strain gauging. (A) Local mono‐arthritis was induced with a systemic injection of mBSA on the first day of loading, followed by a local injection in one knee 1 week later. The mice were loaded for three sessions per week for 2 weeks. Each loading session was accompanied by a subcutaneous injection of NS‐398 (5 mg/kg/d) or vehicle. (B) The relationship between peak dynamic load and strain at the gauge site as calculated using linear regression analysis. Values are presented as mean ± SEM (N = 6). (C) Representative image of the area for epiphyseal and diaphyseal measurement in part of tibia. Supplemental Fig S2. Histological scoring of (A) synovitis, (B) bone erosion, and (C) articular cartilage damage is shown. Wilcoxon signed rank test was performed. Results are shown as median, min. max. to show all points; each group N = 6–8. Supplemental Table S1. Antibodies Supplemental Table. [file JBM4-7-e10751-s001.pdf]

Figure S1

A.

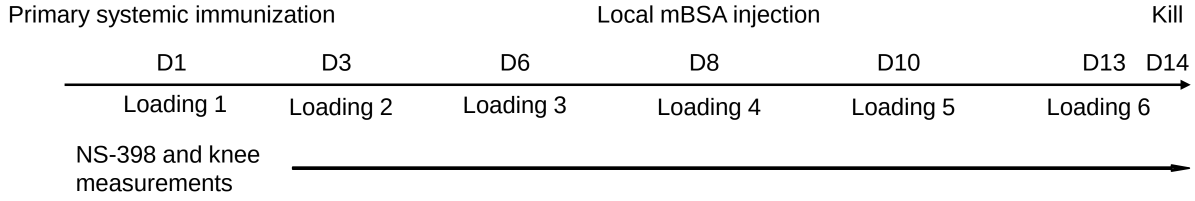

B.

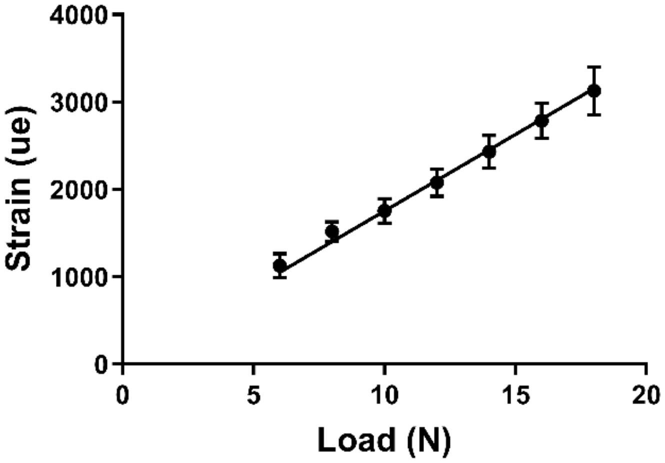

C.

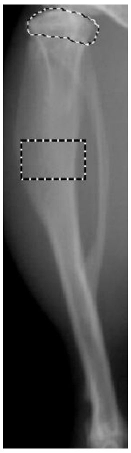

Figure S2

A

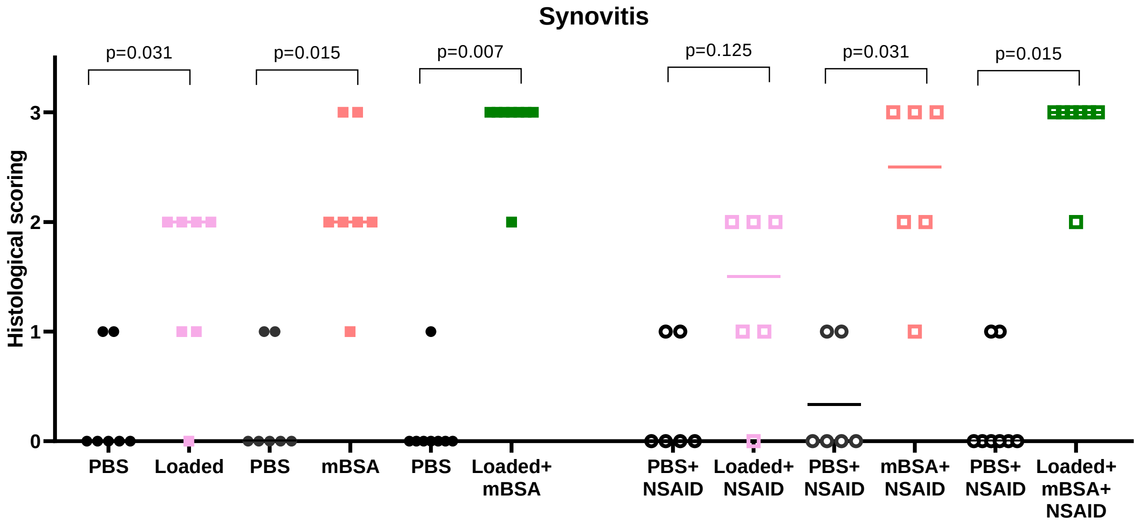
Synovitis
p=0.031
p=0.015
p=0.007
p=0.125
p=0.031
p=0.015
Histological scoring
3
2
1
0
PBS
Loaded
PBS
mBSA
PBS
Loaded+ mBSA
PBS+ NSAID
Loaded+ NSAID
PBS+ NSAID
mBSA+ NSAID
PBS+ NSAID
Loaded+ mBSA+ NSAID

B

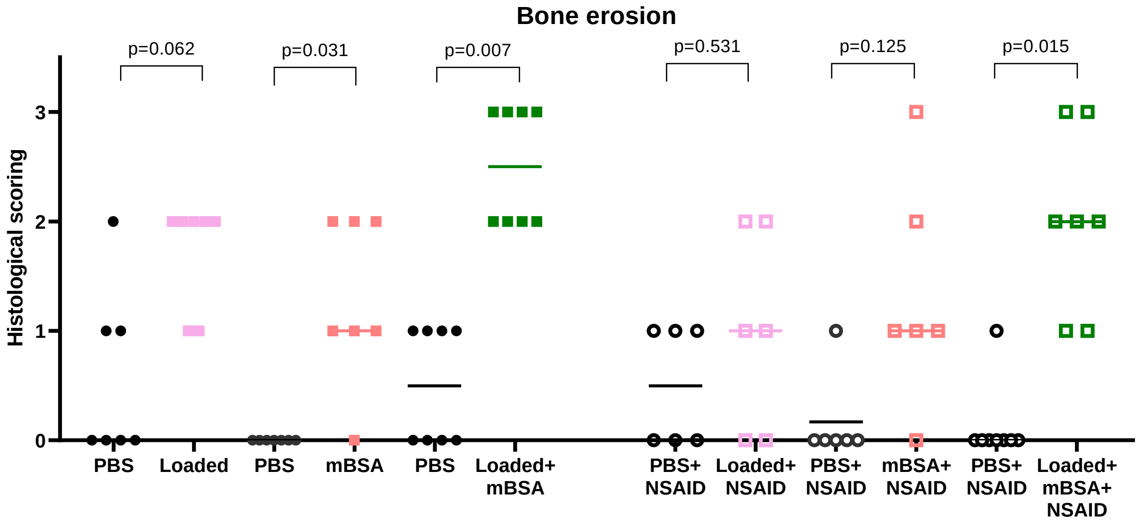
Bone erosion
p=0.062
p=0.031
p=0.007
p=0.531
p=0.125
p=0.015
Histological scoring
3
2
1
0
PBS
Loaded
PBS
mBSA
PBS
Loaded+ mBSA
PBS+ NSAID
Loaded+ NSAID
PBS+ NSAID
mBSA+ NSAID
PBS+ NSAID
Loaded+ mBSA+ NSAID

C

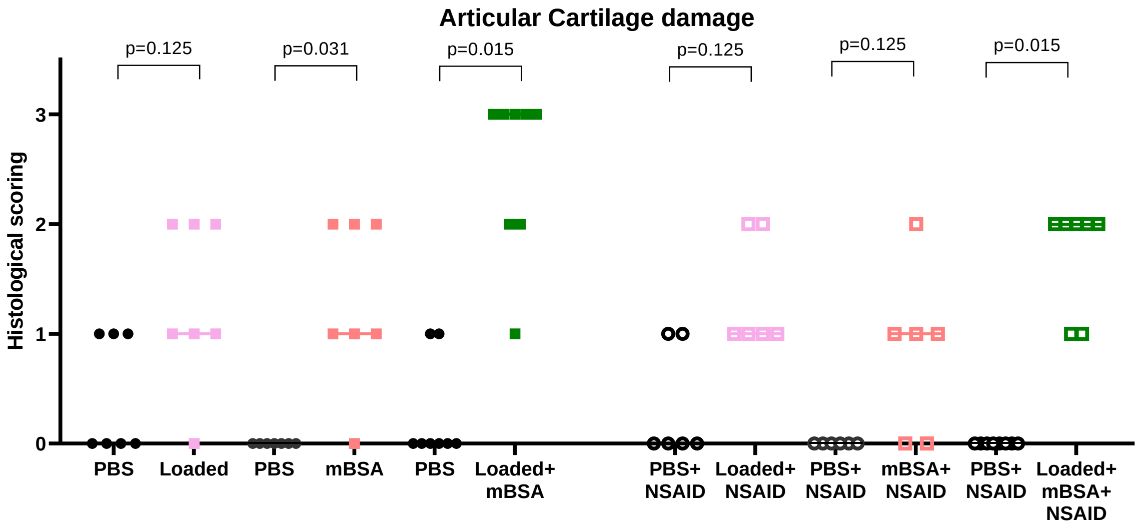

Articular Cartilage damage
p=0.125
p=0.031
p=0.015
p=0.125
p=0.125
p=0.015
Histological scoring
3
2
1
0
PBS
Loaded
PBS
mBSA
PBS
Loaded+ mBSA
PBS+ NSAID
Loaded+ NSAID
PBS+ NSAID
mBSA+ NSAID
PBS+ NSAID
Loaded+ mBSA+ NSAID

Supplemental table 1:
Antibodies supplemental table

Epitope/Antigen or product name	Source and catalog	Host species	Application	Dilution	Application specific details
Mouse Anti cathepsin-K	Agrisera (Sweden) ([28])	Rabbit	IHC	1:300	PBST+ 1.5% NGS, 2 hours, RT
Biotinylated anti-rabbit	DAKO-E0432 (Denmark)	Goat	IHC	1:300	PBST, 30 minutes, RT
HRP conjugated anti mouse	DAKO-P026002-2 (Denmark)	Rabbit	ELISA	1:5000	1 hour, RT
